# Neuromodulation techniques for acute and preventive migraine treatment: a systematic review and meta-analysis of randomized controlled trials

**DOI:** 10.1186/s10194-020-01204-4

**Published:** 2020-12-10

**Authors:** Xavier Moisset, Bruno Pereira, Daniel Ciampi de Andrade, Denys Fontaine, Michel Lantéri-Minet, Jérôme Mawet

**Affiliations:** 1 Service de Neurologie, Biostatistics unit (DRCI), Université Clermont Auvergne, CHU de Clermont-Ferrand, Inserm, Neuro-Dol, 58 rue Montalembert, F-63000 Clermont-Ferrand, France; 2 Department of Neurology, LIM 62 Pain Center, Universidade de São Paulo, São Paulo, Brazil; 3 Department of Neurosurgery, Université Côte Azur, FHU InovPain, CHU Nice, Nice, France; 4 Pain Department, Université Côte Azur, FHU InovPain, CHU Nice, Nice, France– Université Clermont-Auvergne, INSERM, Neuro-Dol, Nice, France; 5 Emergency Headache Center (Centre d’Urgences Céphalées), Department of Neurology, Lariboisière Hospital, Assistance Publique des Hôpitaux de Paris, Paris, France

**Keywords:** Neurostimulation, Remote electrical neuromodulation, Occipital nerve stimulation, Transcutaneous electrical nerve stimulation, Percutaneous electrical nerve stimulation (PENS), Repetitive transcranial magnetic stimulation, Vagus-nerve stimulation, Transcranial direct current stimulation

## Abstract

**Background:**

Several neuromodulation methods exists for migraine treatment. The aim of the present study was to perform a systematic review and meta-analysis of randomized controlled trials (RCTs) focusing on migraine treatment using neurostimulation methods.

**Methods:**

We searched Medline and Embase up to July 1, 2020 for RCTs reporting acute or preventive treatment of migraine with either non-invasive or invasive neurostimulation methods. Two researchers independently assessed the eligibility of the retrieved studies and extracted data. Outcomes for the quantitative synthesis were 2 h pain free for acute treatment and headache days per month for preventive treatment. We performed subgroup analyses by treatment (stimulation method and site of application). Estimates were pooled using random-effects meta-analysis.

**Results:**

Thirty-eight articles were included in the qualitative analysis (7 acute, 31 preventive) and 34 in the quantitative evaluation (6 acute, 28 preventive). Remote electrical neuromodulation (REN) was effective for acute treatment. Data were insufficient to draw conclusions for any other techniques (single studies). Invasive occipital nerve stimulation (ONS) was effective for migraine prevention, with a large effect size but considerable heterogeneity, whereas supra-orbital transcutaneous electrical nerve stimulation (TENS), percutaneous electrical nerve stimulation (PENS), and high-frequency repetitive transcranial magnetic stimulation (rTMS) over the primary motor cortex (M1) were effective, with small to medium effect sizes. Vagus-nerve stimulation, left prefrontal cortex rTMS, and cathodal transcranial direct current stimulation (tDCS) over the M1 had no significant effect and heterogeneity was high.

**Conclusion:**

Several neuromodulation methods are of potential interest for migraine management, but the quality of the evidence is very poor. Future large and well-conducted studies are needed and could improve on the present results.

**Supplementary Information:**

The online version contains supplementary material available at 10.1186/s10194-020-01204-4.

## Key findings

Remote electrical neuromodulation (REN) seems effective for acute treatment.

Invasive occipital nerve stimulation (ONS) is effective for chronic migraine prevention.

Supra-orbital transcutaneous electrical nerve stimulation (TENS), percutaneous electrical nerve stimulation (PENS), and high-frequency repetitive transcranial magnetic stimulation (rTMS) over the motor cortex (M1) are effective for migraine prevention.

## Introduction

According to the Global Burden of Disease study, more than 1 billion people were suffering from migraine in 2016, making migraine one of the most prevalent neurological disorders worldwide [1]. Migraine is even the first cause of disability worldwide among women between the age of 15 and 49 years. Although many pharmacological treatments can be proposed, their efficacy and safety are only partial [2]. Despite the arrival of new drugs, such as those targeting CGRP, certain patients still do not experience optimal relief and other treatments are needed. Moreover, certain patients are reluctant to use pharmacological treatments.

Neuromodulation can be defined as an intervention (drug or procedure) that potentiates or inhibits the transmission of a nerve impulse but is not the actual means of transmission itself [3]. Many medical and interventional techniques can be categorized as neuromodulatory according to this nomenclature and the classification of the various neuromodulatory approaches frequently varies. One frequently used approach is to identify the nervous structure that is targeted (e.g., peripheral nerves, spinal cord, cortex) and then classify the techniques into invasive and noninvasive.

Several non-invasive and even invasive neurostimulation methods have been proposed for acute or preventive migraine treatment. This technology is emerging as a practical and safe alternative to conventional pharmacological treatment of migraine, especially for sensitive patient populations (e.g., pregnant women or adolescents) or those affected by poor tolerability or lack of efficacy of pharmacological approaches. Additionally, non-pharmacological neuromodulatory approaches may even be cost-effective in certain instances [4]. To date, no systematic review combined with meta-analysis of randomized controlled trials (RCTs) on all neurostimulation methods has been published. Thus, we aimed to provide an original comprehensive systematic review and meta-analysis of RCTs focusing on neurostimulation techniques for migraine treatment.

## Methods

The study was submitted to PROSPERO on April 23rd, 2020 (CRD42020181494).

### Procedures

The systematic literature review was performed according to the Preferred Reporting Items for Systematic Reviews and Meta-Analyses (PRISMA) statements [5] and we used a standardized review and data extraction template (Additional file 1). RCTs published as full reports in peer-reviewed journals from inception to May 20, 2020 were identified using PubMed/MEDLINE and Embase. The same databases were further updated up to July 1, 2020.

The target population was patients of any age, including children, with migraine according to the international classification of headache disorders (ICHD) criteria [6]. The migraine conditions considered included both episodic and chronic migraine, either with or without aura. Studies focusing on other headache types, especially tension-type headaches or cluster headaches, were excluded.

The inclusion criteria were in accordance with current methodological guidelines and previous systematic reviews [7–11]: randomized design; comparison with a control group (placebo or comparator) over a follow-up period of at least 4 weeks for preventive treatments (i.e. at least 4 weeks of assessment of the efficacy of active treatment and of comparator in the case of crossover trials) and at least 2 h for acute treatment; at least 10 patients per group; pain assessed as a primary or secondary outcome (number of headache days/month for preventive treatment; proportion of pain-free patients 2 h after treatment for acute treatment); and studies published as full reports. As achieving blinding can be difficult or even impossible for several neurostimulation techniques, such a design was not required.

### Evidence summary and reporting

We used the Grading of Recommendations Assessment, Development and Evaluation (GRADE) system to assess the risk of bias for individual studies and groups of studies.

### Risk of bias in individual trials

Two review authors (XM and JM) independently assessed the risk of bias for each study using the GRADE system for individual studies. As proposed in previous systematic reviews [10, 11], we assessed the following for each study: (1) random sequence generation (checking for possible selection bias) and allocation concealment, (2) blinding of participants (checking for possible performance bias), (3) blinding of evaluators, (4) incomplete outcome data (checking for possible attrition bias due to the amount, nature, or handling of incomplete outcome data), (5) statistical methods, (6) sample size calculation, (7) selective reporting, (8) carryover effect, evaluated for crossover studies and noted as “not applicable” for parallel group studies, and (9) the presence of any other bias evaluated separately, including, in particular, small sample size.

The overall quality of the study was considered “very high” if no bias was noted, “high” if there were only one or two biases, “moderate” if there were three or four, and “low to very low” if there were five or more, as proposed elsewhere [11].

### Outcome measures

We conducted a meta-analysis for this systematic study. An overall meta-analysis was not appropriate as the various techniques have very different mechanisms of action and dosing. Thus, metanalyses were performed grouping stimulation techniques by type.

We report the difference in headache days/month for preventive treatments and/or the responder rate for a 50% decrease in headache days (or alternatively, a 30% decrease). The number of headache days per month was chosen instead of the number of migraine days per month as this measure was more consistently available. For acute treatment, we report the proportion of pain-free patients at 2 h, as recommended [9], and/or, alternatively, the absolute difference in pain reduction at 2 h and/or pain relief at 2 h. Efficacy was assessed on the basis of effect size or absolute difference between active and placebo or sham, or number needed to treat (NNT) (Additional file 2 for acute treatment and 3 for preventive treatment). Effects on other outcome measures, such as quality of life, need for rescue medication, and Patient Global Impression of Change score, were reported when relevant (for example, if no efficacy was observed for the primary outcome measure), but were reported as secondary outcomes. Serious and common (> 10% incidence) reported adverse events and dropout due to adverse effects were recorded (Additional files 2 and 3).

Statistical analysis was performed using Stata software (version 15, StataCorp, College Station, US). The meta-analysis accounted for between- and within-study variability. The non-independence of data due to study effect was addressed using random-effects models [12] to estimate relative risks (RR) and standardized paired mean differences (SMD) and their 95% confidence intervals rather than the usual statistical tests. Means and standard-deviations (SD) were compiled when available or estimated using the approach of Hozo et al. [13] when medians and interquartile ranges were reported. The standard-deviation of the paired differences between baseline (T0) and last follow-up (T1) was estimated using the formula: √((*SD*^2^*T*0 + *SD*^2^*T*1) − (2 ∗ *SDT*0 ∗ *SDT*1)). The SMD is used as a summary statistic in meta-analysis when the studies all assess the same outcome but measure it in a variety of ways. It expresses the size of the intervention effect in each study relative to the variability observed in that study. SMD were interpreted according to Cohen rules: trivial < 0.2 ≤ small < 0.5 ≤ medium < 0.8 ≤ large [14]. The aforementioned statistical approaches were used for sub-group analyses according to the stimulation techniques and target. Sub-group meta-analysis were possible when at least two studies with a similar design were available. Heterogeneity in the study results was assessed by forest plots and the I^2^ statistic, which is the most commonly used metric for measuring the magnitude of between-study heterogeneity and is easily interpretable. I^2^ values range between 0% and 100% and are typically considered low for 25%, modest for 25% to 50%, and high for > 50% [15]. Publication bias was assessed using funnel plots, Egger’s tests, and confidence intervals.

## Results

**Fig. 1:**
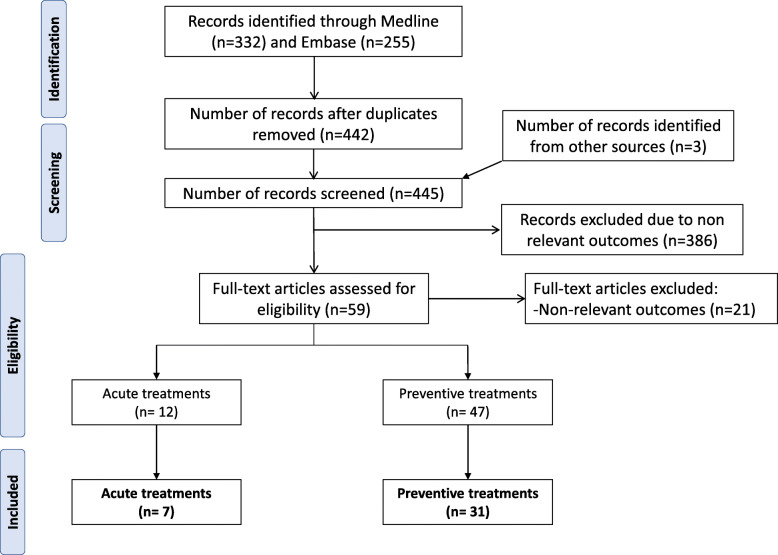
Flow diagram of article selection

The results of the database and registry search are shown in Fig. 1. We included 38 published articles in the qualitative analysis, seven focusing on acute treatment and 31 on preventive treatment. The complete list of studies is available as online supplementary references and the characteristics of each study are summarized in Additional files 2 and 3.

### Acute migraine treatment

**Fig. 2:**
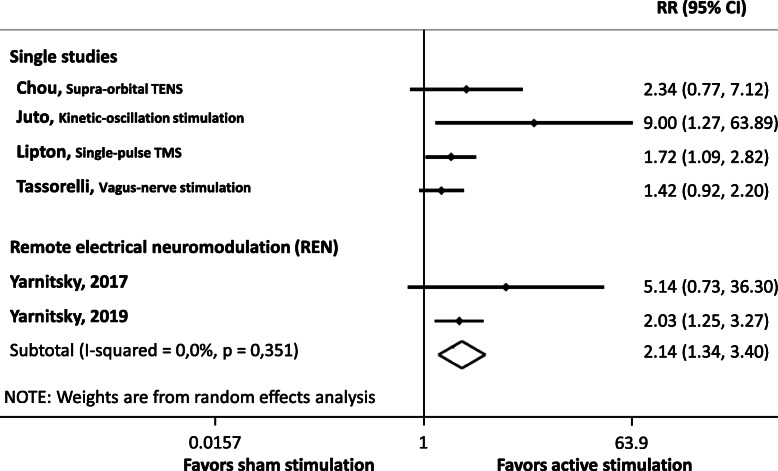
Forest plot of the six studies included in the quantitative synthesis for acute migraine treatment. The common outcome for these six studies was the proportion of patients who were pain free after 2 h in the active treatment and control groups (the higher the relative risk, the more effective the treatment). All references for studies in this figure are listed in the online supplementary references. RR: relative risk, TENS: transcutaneous electrical nerve stimulation, TMS: transcranial magnetic stimulation. The study by Hokenek et al. of 2020 [16] (supra-orbital TENS), was not included in the quantitative analysis

The effect sizes for each study are presented in Fig. 2. Sub-group meta-analysis was possible for remote electrical neuromodulation (REN; 2 studies) only.

#### Non-invasive peripheral neurostimulation

Two studies conducted by a single group and including one very high-quality study were positive for two-hour pain relief for remote electrical neuromodulation (REN) on the arm used as an acute treatment [17, 18]. The NNT was 5 in the smaller study and 3.6 in the larger higher-quality study. Pooled analysis confirmed the positive effect of REN, with a large effect size (RR = 2.14, 95%CI: 1.34–3.40).

Two studies of moderate [16] and very high [19] quality that tested supra-orbital transcutaneous electrical nerve stimulation (TENS) for acute treatment were positive for their primary outcomes and most secondary outcomes, although opposite results were reported for being pain free at 2 h. It was not possible to conduct a pooled analysis of these two studies as the proportion of responders was not given in the study by Hokenek et al..

One very high-quality trial was negative for acute treatment based on the proportion of pain-free patients at 2 h using vagus nerve stimulation (VNS) with a Gammacore™ device [20].

#### Invasive peripheral neurostimulation

A single trial tested a minimally invasive technique. This high-quality trial evaluated the efficacy of mechanical neurostimulation at 68 Hz close to the sphenopalatine ganglion for 30 min and was positive, 50% of the patients being pain free at 2 h following active stimulation versus 8% with the sham [21].

#### Non-invasive central neurostimulation

A single very high-quality study tested single-pulse transcranial magnetic stimulation (TMS) for acute treatment of migraine with aura [22]. The study was positive for a pain-free response rate after 2 h (NNT = 5.9).

#### Invasive central neurostimulation

Based on our inclusion criteria, we were unable to obtain data for the use of invasive central neurostimulation techniques for migraine treatment.

### Preventive migraine treatment

**Fig. 3:**
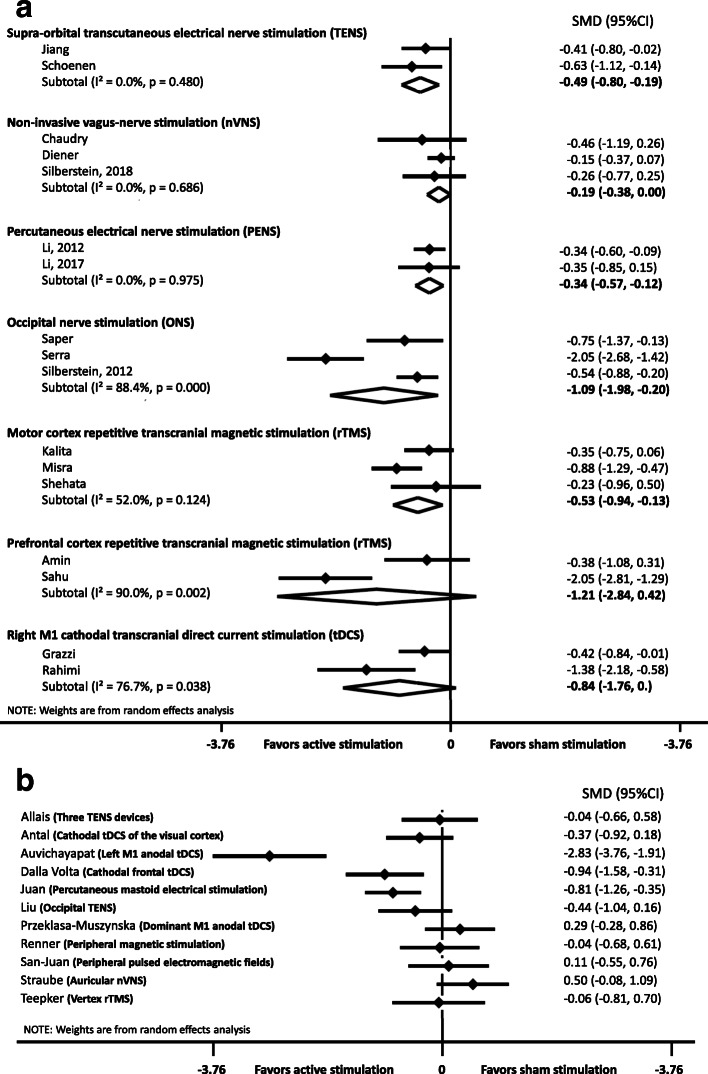
Forest plot of the 27 studies included in the quantitative synthesis for preventive migraine treatment (Panel **a**, at least two studies. Panel **b**, single studies). The common outcome was the reduction of headache days in the active treatment and control groups (the higher the reduction, the more effective the treatment). Studies using the same techniques are pooled together. All references for studies in this figure are listed in the online supplementary references. SMD: standardized mean difference, tDCS: transcranial direct current stimulation. Three studies were not included in the quantitative synthesis: Allais et al., [23] (3 TENS devices); Bono et al., [24] (occipital TENS); Jia et al., [25] (PENS); Kosari et al., [26] (tDCS over the visual cortex)

The effect size for each study of a preventive migraine treatment are presented in Fig. 3.

#### Non-invasive peripheral neurostimulation

One very high-quality study using a percutaneous mastoid electrical stimulator as preventive treatment for episodic migraine was positive with a large effect size (absolute difference of 2.87 days/month reduction) [27].

One moderate-quality study using peripheral pulsed electromagnetic fields applied to the wrist was negative [28].

Five studies investigated the efficacy of TENS. Two tested supra-orbital TENS for preventive treatment. Among them, one moderate-quality study was positive and was tested as an add-on therapy [29], whereas the other high-quality-study tested TENS alone, showing a nearly significant result and becoming significant when baseline migraine days were considered as a covariate [30, 31]. The pooled analysis confirmed the efficacy of supra-orbital TENS with a small effect size (− 0.494, 95%CI: − 0.799 to − 0.188) and no heterogeneity (I^2^ = 0%). One low-quality study tested the use of three TENS devices at the same time to stimulate the face, cervico-occipital region, and hand. TENS was used 5 days/week for three consecutive weeks and was compared to laser therapy and acupuncture [23] (qualitative analysis only). After 1 month, TENS and laser therapy where more efficient than acupuncture. Finally, two moderate-quality trials investigated the use of occipital TENS as preventive therapy. The first, using 40-Hz stimulation, was negative [32]. Various stimulation parameters were used for the second and 100 Hz was positive relative to sham but less effective than topiramate. It was not possible to pool the results of these two studies, as numerical data were not available for the latter [24].

Three trials (one of moderate quality, one of high quality, and one of very high quality) tested VNS delivered using a Gammacore™ device and were negative [33–35]. The pooled analysis showed an absence of heterogeneity (I^2^ = 0%) and did not demonstrate a significant positive effect of VNS (− 0.187, 95%CI: − 0.379 to 0.004). One high-quality trial tested the use of auricular VNS (Vitos™ device) as a preventive treatment and was also negative [36].

One study tested repetitive peripheral magnetic stimulation to myofascial trigger points of the neck compared to shoulder-muscle stimulation [37]. As such peripheral magnetic stimulation has an impact on peripheral nerves, it was included in this systematic review. There was no between-group difference, although a reduction relative to baseline was noted in both groups.

#### Invasive peripheral neurostimulation

One high-quality study concerned percutaneous electrical nerve stimulation (PENS), which is a minimally invasive form of electrical stimulation that requires the insertion of needles for the duration of the stimulation. This study performed on patients with episodic migraine was positive, with a moderate effect size (absolute difference of 1.5 days/month reduction) [38]. Electro-acupuncture is another way to perform PENS and was tested in two studies, one very high-quality study being negative on the primary outcome but positive on all secondary outcomes [39] and the second, of very low quality, being positive on a composite parameter that included pain intensity, pain duration, attack frequency, accompanying symptoms, and plasma 5-HT levels [25]. The last study was not usable for quantitative synthesis. The pooled analysis favored a positive effect of PENS on various acupoints, with a small effect size (− 0.344, 95%CI: − 0.571 to − 0.116) and the absence of heterogeneity (I^2^ = 0%).

Three studies explored invasive occipital nerve stimulation (ONS), all for chronic migraine. The only high-quality trial was negative for its primary outcome [40] but positive for most secondary outcomes, whereas the two others where positive for their primary outcome [41, 42]. The pooled analysis favored a positive effect of invasive ONS, with a large effect size (− 1.090; 95%CI: − 1.977 to − 0.204) but high heterogeneity between studies (I^2^ = 88%).

#### Non-invasive central neurostimulation

Six studies tested repetitive TMS (rTMS). Two (one of low quality that was negative and one of high quality that was positive) conducted by the same group used high-frequency (10 Hz, 600 pulses) repetitive TMS over the left primary motor cortex (M1) as preventive treatment for chronic migraine [43, 44]. A single session was effective in reducing the number of headache days per month for chronic migraine sufferers, with an absolute difference of 3.2 days/month versus placebo. Three sessions did not provide greater pain reduction than a single session. One low-quality study compared botulinum toxin injection to high-frequency repetitive TMS (10 Hz, 2000 pulses per session) over the left M1, three times a week for four consecutive weeks, with a total follow-up of 12 weeks, on patients with chronic migraine [45]. Repetitive TMS showed no difference from botulinum toxin at weeks 4 and 8 but was less effective at week 12. The pooled analysis of these three studies focusing on high-frequency rTMS over the left M1 favored a positive effect, with a medium effect size (− 0.533, 95%CI: − 0.940 to − 0.126) and moderate heterogeneity (I^2^ = 52%). Two other studies (low and moderate quality) targeting the left dorsolateral prefrontal cortex (DLPFC) were positive for their primary outcomes, using a frequency of 5 Hz in one case [46] and intermittent theta-burst stimulation (iTBS) in the other [47], but the first study was negative for the reduction of headache days. The pooled analysis of these two studies focusing on high-frequency rTMS over the left DLPFC did not favor a positive effect and showed high heterogeneity between studies (− 1.210, 95%CI: − 2.844 to 0.423; I^2^ = 90%). The last study focusing on TMS tested low-frequency repetitive TMS (1 Hz, 1000 pulses) over the vertex for five consecutive days compared to sham stimulation [48]. This low-quality study was negative.

Seven studies tested transcranial direct current stimulation (tDCS), with various targeted areas. Two low-quality trials tested cathodal tDCS over the visual cortex (Oz on the International 10–20 EEG system). In one study, in which sessions were performed three times per week, for three consecutive weeks and compared to sham stimulation, was negative [49]. The second study was claimed to be positive by the authors but the poor quality of the report did not provide the opportunity to verify these results and made it impossible to integrate the results of this last study into the quantitative synthesis [26]. One moderate-quality study investigated the use of left M1 anodal tDCS for 20 consecutive days compared to sham stimulation and was positive, with an absolute difference of 1 day per month [50]. A low-quality study investigated anodal tDCS over the dominant M1 and was positive after 10 sessions applied over 30 days [51]. Another study was a moderate-quality trial comparing cathodal-tDCS over the right M1 or primary sensory cortex (S1) versus sham stimulation for 22 sessions spread over 10 consecutive weeks and was positive for the two active stimulation groups [52]. The difference was very large in both groups, with 12.47 days/month for the M1 and 9.47 for the S1 compared to sham. Another recent moderate-quality study compared anodal, cathodal, and sham tDCS over the right M1, used daily for the 5 days of treatment withdrawal for patients with chronic migraine associated with medication overuse [53]. There was no significant difference in the number of migraine days per month 12 months later. The pooled analysis of these two studies focusing on cathodal tDCS over the M1 did not favor a positive effect and showed high heterogeneity between studies (− 0.836, 95%CI: − 1.764 to 0.091; I^2^ = 77%). Recently, a moderate-quality study tested the use of five daily sessions of cathodal tDCS over the coolest frontal region and was positive for the number of migraine days, with a large effect size and an absolute reduction of approximately 8 days/month relative to the placebo group [54].

#### Invasive central neurostimulation

Our standardized search and inclusion criteria did not retrieve any studies for invasive central neurostimulation techniques for migraine treatment.

## Discussion

**Fig. 4:**
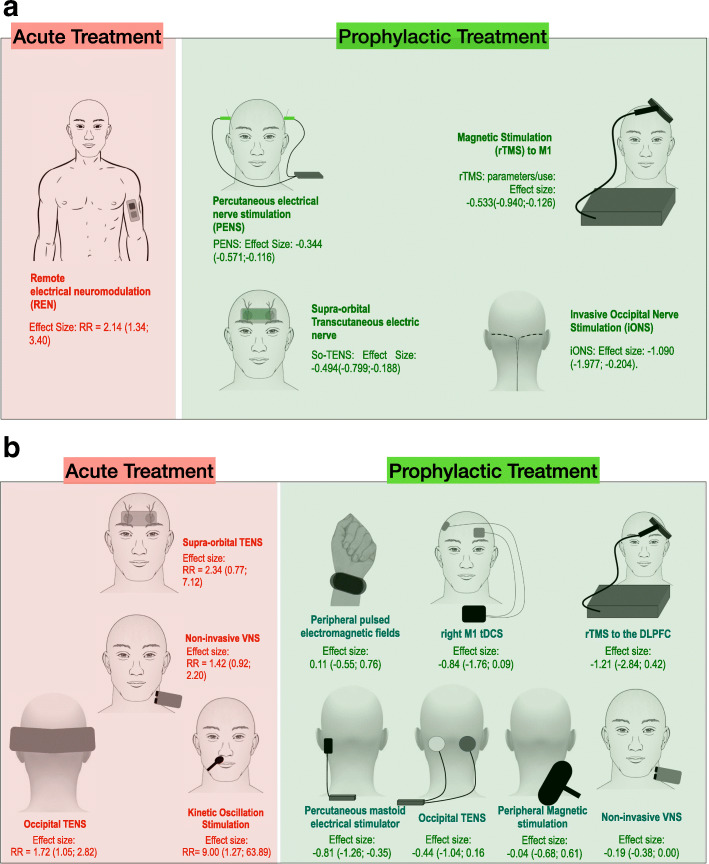
Summary of neuromodulatory techniques included in the meta-analysis. **a**: Techniques found to be effective for acute or preventive migraine treatment according to the meta-analysis (at least 2 includable RCTs). **b**: Other techniques included in the meta-analysis. Effect sizes were noted as relative risk (RR) for acute treatments (the higher the RR, the more effective the method) or standardized mean difference (SMD) for preventive treatments (the more negative the SMD, the more effective the method). 95% confidence intervals were provided (minimal; maximal). rTMS: repetitive transcranial magnetic stimulation, M1: primary motor cortex, TENS: transcutaneous electric nerve stimulation. VNS: vagus nerve stimulation, tDCS: transcranial direct current stimulation Technical details from stimulation modalities cited in figure 4a: **REN**: parameters/use: single 20-45-minute symmetrical biphasic square pulse at 80-200Hz, pulse width of 45-400 μs, up to 40 mA. Main side effects: local unpleasantness (common), numbness in arms and local warmth sensation (rare); [Yarnitsky 2017, Yarnitsky 2019] **PENS**: parameters/use: 0.18-0.32 mm x 25-40 mm needles inserted into unilateral or bilateral acupoints connected to an electro-stimulator [Taiyang EX-HN 5 for bilateral stimulation with the positive pole at the painless side of EX-HN 5 and negative pole at the painful side; for unilateral stimulation Shaoyang - "gallbladder meridian points" were used] 30-minute sessions disperse-dense waves at 2/100Hz, 5 x week for 4-12 weeks. Main side effects: local bleeding (very common), transient leg weakness (rare) [Li H 2012, Li Y 2017] **So-TENS**: parameters/use: as a stand-alone or as an add-on treatment, 30x94 mm adhesive electrode on the forehead, biphasic rectangular pulse width 30 μs, 60 Hz, max 16 mA, 20 min, extended for 3 months. Main side effects: paresthesia and pressure sensation over the electrode spot (common), somnolence (rare); [Jiang 2018, Schoenen 2013]. **rTMS to M1**: parameters/use: Figure-of-eight coil, left M1 at 10Hz, 600-3000 pulses per session, stimulation at 70-80% below rest motor threshold, 1-10 sessions Effect size: -0.533(-0.940;-0.126). Main side effects: scalp discomfort (common), transient drowsiness (rare); [Misra 2013; Shehata 2016; Kalita 2016]. **ONS**: parameters/use: implanted lead (uni- or bilateral) perpendicular to the occipital nerves at the craniocervical junction. Parameter settings were bipolar montage, 50Hz, pulse width 330-450 µsec, bellow 10.5V amplitude. Main side effects: lead migration, local pain over the implanted pulse generator, increase in migraine (common), local infection (rare); [Saper 2010; Silberstein 2012; Serra 2012]

Our systematic review and meta-analyses on non-invasive and invasive stimulation techniques for the preventive treatment of migraine favor a positive effect for invasive ONS, with a large effect size but considerable heterogeneity and a positive effect for supra-orbital TENS, PENS, and high-frequency rTMS over the M1 with small to medium effect sizes (Fig. 4). Practical information for each device is provided in Table 1. Vagus-nerve stimulation was nearly statistically significant but with an effect size below 0.2, which is not clinically relevant. Left prefrontal cortex rTMS and tDCS over the M1 had no significant effect and the heterogeneity between studies was high in both cases. For acute treatment, REN was effective. There are very few techniques that have been evaluated appropriately in at least two randomized controlled trials, precluding any meta-analysis and conclusion in most of the other cases.

**Table 1:** Summary of type of stimulation, targets brand name, manufacturer, practical use and CE/FDA approval for main devices tested for migraine treatment

Type of stimulation	Target	Brand name™	Manufacturer	Practical use A = Acute P = prophylactic	FDA cleared	CE mark
Remote electrical nerve (REN) stimulation inducing conditioned pain modulation	Arm	Nerivio Migra™	Theranica	A	+	+
Invasive electrical stimulation	Great occipital nerve	Quad/Synergy™^a^ Genesis/Eon Mini™^b^	Medtronic St. Jude Medical-Abbott	P	–	–
Transcutaneous electrical nerve stimulation (TENS)	Supra-orbital nerve	Cefaly™	Cefaly technology	P	+	+
High frequency repetitive TMS	Primary motor cortex (M1)	More than 4	More than 4	P	+	+
Percutaneous electric nerve stimulation (PENS)	Shaoyang or Taiyang acupoints	More than 4	More than 4	P	+	+
Non-invasive vagus nerve stimulation	Cervical vagus nerve	GammaCore™ NEMOS/ VITOS™	ElectroCore tVNS Technologies GmbH	A and P	+	+
Transcutaneous electrical nerve stimulation (TENS)	Supra-orbital nerve or occipital nerve	Cefaly™ Headaterm™ Relivion™ + Non-specific TENS devices	Cefaly technology WAT medical Neurolief	A and P	+	+
Single pulse transcranial magnetic stimulation (TMS)	Occipital cortex	SpringTMS™	eNeura	A	+	+
High frequency repetitive TMS	Dorsolateral prefrontal cortex and vertex	More than 4	More than 4	P	+	+
Transcranial direct current stimulation (tDCS)	M1, visual cortex, frontal cortex	More than 4	More than 4	P	+	+

Stimulation methods with positive effect according to pooled results of at least 2 studies included in the present review are presented above the thick line

^a^These devices were used in the studies by Serra and Saper et al.. However, the Synergy™ generator is not manufactured any longer and replaced by the Prime Advanced™ (non-rechargeable) or Intellis™ (rechargeable) generator

^b^These generators are not commercialized any more by St Jude-Abbott and replaced by the Proclaim™ generators

To date, the number of systematic reviews and meta-analyses focusing on neurostimulation procedure for migraine treatment is limited. There was no comprehensive review with analysis of each specific stimulation methods and targets (Table 2) [55–66]. Moreover, certain results of these previous publications are disputable, as meta-analysis pooled results from studies using different stimulation techniques (rTMS and tDCS, or TENS, VNS and PENS) or different targets (M1 and frontal or supra-orbital and occipital).. Indeed, the mode of action of these various stimulation methods are different and target different cortical regions or peripheral nerves [67–69]. In the last well-conducted systematic review [70], the goal was to evaluate the scientific rigor of published studies to allow for proper comparison between devices.

**Table 2:** Summary of the main results of previous systematic reviews and meta-analysis focusing on neuromodulation in migraine treatment

	First author	Date	Journal	Topic	Main conclusion
rTMS / tDCS	Fregni	2020	Int J Neuropsychopharmacol	tDCS in neurological and psychiatric disorders	tDCS is probably effective (Level B) for migraine
	Baptista	2019	Pain reports	Latin American and Caribbean consensus on rTMS and tDCS for chronic pain management	Level B recommendation for anodal tDCS over M1 or Oz/Cz tDCS. Level B recommendation for high-frequency rTMS over M1
	Feng	2019	Headache	rTMS and tDCS	Excitatory M1 stimulation showed significant effects on reducing headache intensity and frequency with a large effect size. Excitatory DLPFC stimulation showed a significant effect on the headache intensity with a large effect size but no reduction of frequency of headache attacks.
	Stilling	2019	Headache	TMS and tDCS for the treatment of headache	rTMS has moderate evidence that it contributes to reductions in headache frequency, duration, intensity, abortive medication use, depression, and functional impairment.
	Shirahige	2016	Headache	rTMS and tDCS	tDCS reduced migraine attacks frequency (SMD: −0.75; 95% CI: −1.25 to − 0.24; *P* = .004). There was no effect of rTMS.
TMS	Lan	2017	J Headache Pain	Efficacy of single-pulse TMS in randomized controlled trials	Single-pulse TMS is effective for the acute treatment of migraine with aura after the first attack. The efficacy of TMS on chronic migraine was not significant.
ONS	Cadalso	2018	J Oral Facial Pain Headache	ONS in intractable primary headache disorders	3 RCTs: significant reduction of headache days per month (difference = −3.061; 95% confidence interval [CI] = −5.162 to −0.961; P = .004) compared to sham
	Yang	2016	Pain pratice	ONS for migraine	Results from 4 retrospective studies and 1 case series indicated that ONS significantly reduced the number of days with headache in patients with migraine. However, the evidence of ONS efficacy established by 5 RCTs was limited.
	Chen	2015	Plos One	ONS for chronic migraine	3 multicenter RCTs: mean reduction of 2.59 days (95% CI 0.91 to 4.27, I2 = 0%) of prolonged, moderate to severe headache per month at 3 months compared with a sham control.
VNS	Lai	2020	Neuromodulation	Cervical non-invasive VNS for migraine and cluster headache	No significant differences in headache days reduction (SMD = −0.159; 95% CI, − 0.357 to 0.04; *p* = 0.117) between nVNS and sham-device treatment.
TENS	Stanak	2020	J Neurol Sci	Impact of TENS on prevention and acute treatment of episodic and chronic migraine	Reduction of migraine attacks (0.67 less migraine attacks per month), migraine days (1.74 less migraine days per month), headache days (2.28 less headache days per month). Concerning acute treatment, significant reduction of pain at 1/2/24 h post-acute treatment.
	Tao	2018	J Headache Pain	TENS in randomized controlled trials for migraine	Significant reduction of monthly headache days (SMD: −0.48; 95% CI: − 0.73 to - 0.23; *P* < 0.001)

*M1* primary motor cortex, *ONS* occipital nerve stimulation, *RCT* randomized control trial, *rTMS* repetitive transcranial magnetic stimulation, *SMD* standardized mean difference, *tDCS* transcranial direct current stimulation, *TENS* transcutaneous electrical nerve stimulation, *VNS* vagus nerve stimulation

Among tested devices, several are already approved for clinical use, such as the TENS device, called Cefaly™, which is CE marked and FDA cleared. Based on current evidence, it appears to be useful for preventive treatment and possibly acute treatment. The single-pulse TMS device, called SpringTMS™, is also CE marked and FDA approved and appears to be useful for the acute treatment of migraine with aura. For REN, the Nerivio Migra™ is also CE marked and FDA cleared and is useful for acute treatment. Many devices are CE marked and FDA cleared for repetitive TMS and tDCS but patient access is still limited in many countries. To date, the FDA has not cleared an invasive ONS device for the treatment of headache. The St. Jude Medical/Abbot devices (Eon, Genesis) received CE mark approval in Europe for the treatment of chronic migraine in 2012 but the CE mark was rescinded a few years later. Consequently, no ONS device is currently CE marked for migraine. The VNS stimulator Gammacore™ is also CE marked and FDA cleared but cannot be recommended for migraine treatment based on the presented data.

Several techniques are quite similar in terms of mode of action. Such is the case for occipital TENS, occipital PENS, and invasive ONS [68]. In these three instances, electrical stimulation is delivered close to the great occipital nerve and the trigemino-cervical convergence is considered to provide pain relief in the cephalic area in a referred pain pattern area. Thus, these three techniques should have comparable efficacy and one study proposed that the response to occipital TENS could be predictive of the long-term response to ONS, but very few migraine patients were included in this study [71]. There is no clear interest of PENS or occipital nerve blocks to predict ONS efficacy [72, 73]. In the present review, the trend was the same for the three techniques and, as expected, the effect size appears to be larger for invasive techniques, especially ONS, which allows continuous and prolonged stimulation. On the contrary, non-invasive brain stimulation techniques, i.e. rTMS and tDCS, act very differently and provide different results in other chronic pain conditions [74]. To date, these various techniques have not been directly compared in migraine.

There are recommendations for rTMS [75], which are inconclusive for migraine. Compared to the previous work, we included one additional study in the present meta-analysis, which used botulinum toxin as a control group and did not show a significant difference. Our quantitative synthesis favors a positive effect but it is important to note that the two positive studies were conducted by a single group. Concerning the DLPFC target, two new studies have been published since the two conflicting studies discussed in the recommendations but were not eligible (less than 10 patients per study arm in both cases) for the present review [76, 77]. The long-term RCT conducted by Conforto et al. was negative [77]. Our quantitative synthesis does not favor an effect of such stimulation.

For acute treatment, REN is effective, and although one multicentred double-blind sham-controlled trial is included (very-high quality study), it must be noted that the two studies included in the quantitative synthesis were led by a single group. This technique is based on the use of conditioned pain modulation (CPM). CPM can be summarized as “pain inhibits pain”. Since the use of one pain to inhibit another is not clinically appealing, these authors tested nonpainful conditioning, as it has been shown that robust nonpainful conditioning stimuli are sufficient in many cases to inhibit pain. If the results of these two studies are confirmed by an independent group, REN will definitely be an interesting option for acute migraine treatment.

### Limitations

Our review had several limitations. First, our meta-analysis was based on a very limited number of articles for each study sub-group and the estimation of effect size may not be properly powered. Hence, our conclusions should be interpreted with caution. Further research is very likely to have a large impact on the confidence in the estimated effect. Even within the subgroups, in which the same technique and target were tested, the parameters could be quite different in terms of stimulation intensity and the number of sessions, again limiting the reliability of our estimates. Second, although the included studies only considered migraine patients, we cannot exclude the presence of confounding factors due to migraine frequency at baseline and potential associated medication overuse or the presence of overlapping headache disorders among the included subjects, which could affect the validity of our results. Third, the methodological quality of the included studies was heterogeneous, with only 12/38 studies being of high or very high quality in the present review, as shown in previous neuromodulatory reviews for chronic pain in general [57]. Moreover, it is often difficult to obtain proper blinding in studies involving neurostimulation devices that usually induce paresthesia. Finally, the follow-up period was generally relatively short and thus long-term benefits of neuromodulation techniques are yet to be proven. Longer well-conducted studies are still needed.

## Conclusion

Many neurostimulation methods have been proposed for both preventive and acute migraine treatment and there is evidence for the utility of such treatments, especially for patients that have contra-indications or intolerance to usual drugs. Although several techniques and devices appear to be effective (invasive ONS, supra-orbital TENS, PENS, and high-frequency rTMS over the M1 as prophylactic treatments; remote electrical neuromodulation for acute treatment), larger well-conducted studies are still necessary for most to confirm their efficacy and determine their true effect sizes.

## Supplementary Information

**Additional file 1:.** Search criteria and eligibility criteria.

**Additional file 2.**

**Additional file 3.**

## Data Availability

All data generated or analysed during this study are included in this published article and its supplementary information files.

## Abbreviations

CPM: Conditioned pain modulation
DLPFC: Dorsolateral prefrontal cortex
GRADE: Grading of Recommendations Assessment, Development and Evaluation
ICHD: International classification of headache disorders
iTBS: Intermittent theta-burst stimulation
M1: Primary motor cortex
NNT: Number needed to treat
ONS: Occipital nerve stimulation
PENS: Percutaneous electrical nerve stimulation
RCT: Randomized controlled trial
REN: Remote electrical neuromodulation
RR: Relative risk
rTMS: Repetitive transcranial magnetic stimulation
S1: Primary sensory cortex
SD: Standard-deviation
SMD: Standardized paired mean differences
tDCS: Transcranial direct current stimulation
TENS: Transcutaneous electrical nerve stimulation
TMS: Transcranial magnetic stimulation
VNS: Vagus nerve stimulation
