# Supplementary material for: Neuromodulation techniques for acute and preventive migraine treatment: a systematic review and meta-analysis of randomized controlled trials
Source: J Headache Pain. 2020 Dec 10;21(1):142. doi: 10.1186/s10194-020-01204-4 (PMC7726868; doi:10.1186/s10194-020-01204-4)
Supplement: Supplementary file 1 — Additional file 1:. Search criteria and eligibility criteria. [file 10194_2020_1204_MOESM1_ESM.docx]

**Additional file 1: Search criteria and eligibility criteria.**

**Search was performed on May 20^th^, 2020 using these equations:**

﻿Pubmed / Medline:

((("migraine disorders"[MH] OR "migraines"[TW] OR "migraine"[TW] OR "migrainous"[TW] OR "migrane"[TW] OR "typical aura without headache"[TW] OR "sick headache"[TW] OR "sick headaches"[TW] OR "migrainosus"[TW] OR "vascular headache"[TW]))

AND

("electric stimulation therapy"[MH] OR "electrotherapy"[TW] OR stimulation*[TW] OR "transcutaneous electric nerve stimulation"[MH] OR "tens"[TW] OR "neuromodulation"[TW] OR "electrostimulation"[TW] OR "electroanalgesia"[TW] OR "electroacupuncture"[TW] OR modulation*[TW] OR "Implantable Neurostimulators"[Mesh:NoExp] OR "neurostimulator"[TW] OR "neurostimulators"[TW]))

AND

((randomized controlled trial[pt] OR controlled clinical trial[pt] OR randomized[tiab] OR placebo[tiab] OR clinical trials as topic[mesh:noexp] OR randomly[tiab] OR trial[ti]))

AND

Filters: English, French

Embase:

('migraine'/exp OR 'sick headache')

AND ('electrotherapy'/exp OR 'nerve stimulator'/exp OR 'electroanalgesia'/exp)

AND ('controlled clinical trial'/de OR 'double blind procedure'/de OR 'randomized controlled trial'/de OR 'randomized controlled trial topic'/de)

AND ([english]/lim OR [french]/lim)

**Eligibility criteria**

Patients with migraine

All types of neuromodulation or neurostimulation intervention

Presence of a control group (placebo or other treatment)

Follow-up of at least 3 weeks

Articles in English or French

Articles published in peer-reviewed journals

**Non eligibility criteria**

Less than 10 patients per treatment group

Studies published as abstract only

Case reports

Retrospective studies

Uncontrolled observational studies

Article on cluster headache, tension-type headache or other primary or secondary headache.
